# On the use of immune checkpoint inhibitors in patients with viral infections including COVID-19

**DOI:** 10.1136/jitc-2020-001145

**Published:** 2020-07-01

**Authors:** Thilo Gambichler, Judith Reuther, Christina H Scheel, Jürgen Christian Becker

**Affiliations:** 1 Skin Cancer Center, Department of Dermatology, Ruhr-University Bochum, Bochum, Germany; 2 Institute of Stem Cell Research, Helmholtz Center Munich, Neuherberg, Germany; 3 Translational Skin Cancer Research, German Cancer Consortium (DKTK), Dermatology, University Duisburg-Essen, Essen, Germany; 4 German Cancer Research Center (DKFZ), Heidelberg, Germany

**Keywords:** immune evation, tumor escape, T-lymphocytes, vaccination, review

## Abstract

The present review summarizes up-to-date evidence addressing the frequently discussed clinical controversies regarding the use of immune checkpoint inhibitors (ICIs) in cancer patients with viral infections, including AIDS, hepatitis B and C, progressive multifocal leukoencephalopathy, influenza, and COVID-19. In detail, we provide available information on (1) safety regarding the risk of new infections, (2) effects on the outcome of pre-existing infections, (3) whether immunosuppressive drugs used to treat ICI-related adverse events affect the risk of infection or virulence of pre-existing infections, (4) whether the use of vaccines in ICI-treated patients is considered safe, and (5) whether there are beneficial effects of ICIs that even qualify them as a therapeutic approach for these viral infections.

## Introduction

Cancer cells may escape immune surveillance through a variety of mechanisms, including the activation of immune checkpoint pathways that serve to suppress immune responses against tumor cells. Immune checkpoint inhibitors (ICI) boost antitumor immune responses by interrupting coinhibitory signaling pathways to promote immune-mediated killing of tumor cells. The introduction of ICI in 2011 for therapy has been a revolutionary milestone in the management of many solid cancers and hematological malignancies (eg, melanoma, Merkel cell carcinoma, squamous cell carcinomas, colorectal cancer, renal cell carcinoma, urothelial cancer, Hodgkin lymphoma).^1–3^ Currently, antibodies targeting three different inhibitory checkpoint proteins are approved as first-line, second-line or third-line treatments for various types of malignancies: cytotoxic T lymphocyte antigen-4 (CTLA-4; ipilimumab), programmed cell death protein-1 (PD-1; pembrolizumab, nivolumab, cemiplimab), and programmed cell death protein ligand-1 (PD-L1; durvalumab, atezolizumab, avelumab).^1–4^

Full activation of T lymphocytes predominantly depends on several different signals. Indeed, T lymphocyte activation is regulated both by costimulators and coinhibitors known as immune checkpoints.^5^ Antigen-major histocompatibility complex (MHC) and T cell receptor (TCR) binding associated with the activation of costimulatory receptors (ie, CD28) enables T lymphocytes to proliferate, differentiate and migrate toward specific antigens. By contrast, when antigen-MHC and TCR binding is associated with signaling of coinhibitory receptors (ie, CTLA-4), T cell activation will be suppressed. CTLA-4 is not expressed in naïve T lymphocytes, but is quickly induced on T cell activation. Importantly, CTLA-4 predominantly regulates the amplitude of T cell activation during the early priming phase in lymphoid organs.^1 3 5^ The binding of CTLA-4 to B7 proteins is in direct competition with CD28 costimulatory signals, and the ratio between CD28 and CTLA-4 binding determines activation of T lymphocytes versus anergy, and represents an important mechanism in the prevention of excessive immune responses. Hence, the main task of CTLA-4 is to stop autoreactive T lymphocytes at the initial stages of activation, predominantly in lymphoid tissues, to prevent autoimmunity.^5^ Thus, it is not surprising that it is also expressed on regulatory T cells (Tregs). Similar to Tregs, PD-1 plays an important role in limiting immune responses in peripheral tissues. The interaction of PD-1 with its ligands (PD-L1/2) inhibits T cell proliferation and cytokine secretion mediated by TCRs.^1–3 5^ The PD‐1 receptor is physiologically expressed by activated T lymphocytes, B lymphocytes, monocytes, natural killer (NK) cells, and Tregs. PD‐L1 is expressed on several cells, including tumor cells and some host cells such as myeloid, lymphoid and epithelial cells. The interaction between PD‐1 and PD‐L1 blocks CD8+ cytotoxic T cell proliferation and survival, leads to apoptosis of tumor‐infiltrating lymphocytes, and promotes differentiation of CD4+ T lymphocytes into Tregs.^1 5 6^ Most cancer cells possess the ability to express inhibitory ligands such as PD-L1, for example, in response to interferons (IFN). This process can limit normal anticancer immune responses, thus assisting in immune escape. Hence, ICI do not result in killing tumor cells directly but enhance or restore immune responses and endogenous antitumor activity.^1–6^

Exhaustion of T lymphocytes is the most important factor contributing to weakened T cell activity against both cancer and infectious agents. Notably, T cell exhaustion is a distinguishing feature of many chronic viral infections such as HIV and hepatitis B virus (HBV) infection. Indeed, T cell exhaustion was first described in the context of chronic infections.^7 8^ In the following, T lymphocytes with a similar phenotype were also detected in the tumor microenvironment.^2 7–9^ Exhausted T lymphocytes are functionally characterized by a loss of interleukin 2 (IL-2) production, impaired proliferation, diminished cytotoxicity, and altered production of proinflammatory cytokines.^2 7–9^ Moreover, the overexpression of immune checkpoint receptors, including PD-1 and CTLA-4, is a characteristic. Given the similarities between the immune response to cancer and chronic infections, one may hypothesize that the use of ICI should not be harmful for tumor patients with infections or may even provide a benefit. However, as of yet, tumor patients with existing viral infections are excluded from participation in many treatment protocols for ICI.^7 8^ With respect to acquired infectious diseases during ICI treatment, no increased risk was observed in clinical studies.^1–4 7 8^ However, ICI treatment frequently results in activation of autoreactive T lymphocytes and disturbances in immune tolerance thereby causing autoimmune‐like/inflammatory side effects. These are summarized as immune‐related adverse events (irAEs) and include autoimmune colitis, pneumonitis, hypophysitis, hepatitis, thyroiditis and so on.^10–12^ Since irAEs may require immunosuppressive therapy, including high-dose corticosteroids and/or tumor necrosis factor (TNF)-α blockers, the risk of infection or reactivation of chronic or latent viral infections (eg, HBV or hepatitis C virus (HCV)) may be secondarily increased.^10–12^ In this respect, it should also be noted that much of the morbidity of persistent viral diseases is caused by collateral damage caused by the chronic reactive inflammation associated with the inability of viral clearance; both may be boosted by ICI therapy. In this review, we present information on the pros and cons of using ICI in patients with viral infections including COVID-19.

## HIV infection

Antiretroviral therapy has significantly decreased the incidence of AIDS and thus the mortality of HIV infection. However, complete eradication of HIV with antiviral agents has not yet been achieved, presumably because HIV persists in cellular reservoirs. The major HIV reservoir is a small pool of latently infected resting memory CD4+ lymphocytes carrying an integrated form of the viral genome that lacks the ability to produce viral proteins.^13^ In HIV-infected subjects receiving highly active antiretroviral therapy (HAART), inhibitory checkpoint proteins such as PD-1 are expressed on persisting infected T cells. Indeed, there is a wealth of evidence that high expression of PD-1 on CD4+ lymphocytes clearly correlates with HIV persistence.^8 13^ However, different inhibitory checkpoint proteins are differentially expressed by T cell subtypes; for example, PD-1 expression is increased in memory T cells and Tregs, whereas CTLA-4 is highly expressed in both memory T cells and Tregs.^8 14^ Interestingly, the frequency of PD-1 expression on CD4+ and CD8+ lymphocytes appears to strongly correlate with disease outcome. Specifically, in untreated patients with HIV, high PD-1 expression has been shown to correlate with a decrease in CD4+ T lymphocytes during both acute and chronic infection.^7 8 15^ Similar to PD-1 upregulation, overexpression of CTLA-4 on CD4+ T lymphocytes more frequently correlates with progressive disease.^7 8 16^

HIV persistence can be reversed by ICI in vitro,^7 8 17^ and in preclinical animal models, T cell exhaustion is ameliorated by PD-1 blockade.^18^ Together, the above discussed pathomechanism and preliminary experimental data warrant studies regarding safety and efficacy of ICI in HIV-infected patients. Interestingly, even though patients living with HIV on HAART have a life expectancy similar to the general population, these patients still have an increased risk to develop cancer.^19 20^ In this context, initial observations on HIV-positive cancer patients treated with ICI are emerging. In a systematic review, of 73 HIV-infected patients who received ICI therapy for advanced cancer, anti-PD-1 monotherapy was the most frequently employed regimen (n=62).^19^ In this cohort, grade III or higher irAEs were observed in 8.6% of patients. HIV remained suppressed in 93% of patients and CD4+ lymphocyte count increased in patients with available pretreatment and post-treatment HIV load and CD4 cell count data, respectively. None of the previous studies reported the occurrence of immune reactivation inflammatory syndrome during ICI therapy.^19^ Similar to the safety profile, efficacy of ICI was favorable with an objective response of 63% in Kaposi sarcoma, 30% in non-small cell lung carcinoma, and 27% in melanoma.^19^ Prompted by these encouraging results, phase I and II trials investigating ICIs in HIV-infected patients with advanced solid tumors and lymphomas are currently conducted.^21 22^ Accordingly, a task force formed by the American Society of Clinical Oncology (ASCO) recently recommended the inclusion of HIV-infected patients in oncology trials, particularly patients with CD4+ counts higher than 350 cells/µL, thus representing a group of patients with intact immunological function and survival comparable with the general population.^23^ Moreover, data from the first clinical trial investigating the safety, tolerability and pharmacokinetics of CTLA-4 inhibition (ipilimumab) in patients with chronic HIV infection in the absence of concurrent malignancies were recently reported.^24^ Although only based on a limited number of patients (n=24), this study did not reveal any safety concerns that would preclude further investigation of using CLTA-4 inhibition to enhance the immune response against HIV. One patient who developed facial palsy received medium dose prednisone; still, no worsening of his HIV infection was observed.^24^ Furthermore, in a randomized, double-blind, placebo-controlled, phase I dose-escalating study testing PD-1 inhibition (nivolumab) in HAART-treated HIV-infected adults without concurrent cancer (n=8), even a single, low-dose infusion appeared to enhance HIV-specific immunity.^25^

In summary, the exclusion of HIV-infected patients from oncology ICI trials appears to be neither supported by evidence from basic research nor early clinical trials. On the contrary, many lines of evidence suggest that ICI could be employed to improve HIV-specific immunity and thus contribute to HIV remission or even possible cure strategies.^23 26 27^ However, the mechanisms regulating HIV persistence are complex and not yet fully understood, leading to the hypothesis that a combined treatment approach including ICI and cytokines will be required to accomplish such complete remissions.^28^

## Hepatitis B and C

Viral caused hepatitis is one of the leading causes of morbidity and death worldwide. HBV and HCV account for the majority of viral-hepatitis-related mortality, mostly attributable to cirrhosis and hepatocellular carcinoma. To date, it is still a challenge to achieve complete HBV clearance or to prevent HCV relapse once directly acting antiviral treatment regimens failed.^29^

One important constraint for the use of ICI in patients with concomitant virus hepatitis for treatment of cancer is the possible occurrence of immune-mediated hepatotoxicity, a frequent irAE caused by ICI. This notion is particularly troublesome, as an immune-mediated hepatitis may pose a significant diagnostic challenge in patients with underlying viral or autoimmune hepatitis.^30–32^ Pu *et al* 33 recently published a comprehensive review on 186 cancer patients with concurrent HBV or HCV infection who had received ICI treatment.^33^ About 20% of patients developed an increase of hepatic transaminases which was higher than those reported in ICI-treated cancer patients without concurrent viral hepatitis.^33^ All grade 3 or 4 toxicities were reversible by means of antiviral treatment or corticosteroids without necessitating a discontinuation of ICI. Importantly, no negative influence on infection status was reported in patients receiving corticosteroids.^33^ It should be mentioned, however, that ICI should be withhold once an irAE is encountered requiring immunosuppressive drugs; but, ICI may be resumed once the irAE has resolved. Importantly, the incidence of other adverse events in this particular patient population was not significantly increased when compared with ICI-treated cancer patients without chronic viral hepatitis. Based on a recent publication reviewing the available data on the use of ICI in cancer patients with hepatitis, it is recommended that all patients scheduled to receive ICI should be screened for HBV and HCV, and in patients who are tested positive, prophylactic antiviral treatment is indicated. Unfortunately, however, it is currently unclear for how long the prophylactic treatment should be continued. Primary prophylaxis should also be considered in patients with chronic HBV infection, if not already on treatment. Liver function tests and viral load should routinely be monitored in virus positive patients.^34 35^

It is well known that HBV-specific exhaustion of T cells is maintained by ongoing HBV-antigen stimulation. Furthermore, PD-L1/2 expression, secretion of immunosuppressive cytokines, for example, IL-10 and transforming growth factor (TGF-ß), dysfunction of dendritic cells, enhanced numbers of PD-1+ NK cells, Tregs, and myeloid-derived suppressor cells negatively influence HBV-specific T cell immunity.^6 8 34 35^ Similar to HBV infection, chronic HCV infection is also associated with increased PD-1 and TIM-3 expression as another marker for T cell exhaustion.^36 37^ Although direct-acting antiviral regimens for HCV-infected patients have shown great overall success, thereby placing the need for therapeutic alternatives into perspective, the application of ICI to treat therapy-resistant chronic HCV infection is appealing.^28 29 36 38 39^ Gardiner *et al* 40 recently reported a phase I proof-of-concept trial demonstrating that PD-1 inhibition through nivolumab resulted in prolonged suppression of HCV replication in some patients with chronic infection. Based on these encouraging results, further exploration of PD-1 pathway inhibition is warranted for other chronic viral diseases, possibly in combination with direct-acting antiviral regimens.^40^ However, similar to the situation for HBV infection, the number of clinical trials assessing ICI in chronic HCV infection remain limited.^36 41–43^

Taken together, ICI appears to be safe and effective in cancer patients with concurrent HBV or HCV. Thus, HBV and HCV infection should not contraindicate ICI.^34 35^ Even though the risk of virus reactivation and virus-related hepatotoxicity appears to be low, it is recommended that patients with active HBV or HCV should routinely be monitored and treated with antiviral agents if indicated, in particular in patients receiving immunosuppressive medication for ICI-induced irAE.^33^ Since PD-1 plays a significant role in the natural history of both HBV-induced and HCV-induced hepatitis, there is a rationale for the use of ICI in these conditions. Initial studies indicate that anti-PD-1 treatment is safe in chronic HBV and HCV infection, but further trials are needed to determine whether ICI can be used to gain HBV long-term remission.^36^

## Progressive multifocal leukoencephalopathy

Progressive multifocal leukoencephalopathy (PML) is a rare, often lethal disease of the central nervous system caused by the JC polyomavirus (JCV).^44^ In general, JCV infection is indolent and asymptomatic, but may become symptomatic and fatal in immunocompromised patients, for example, patients infected with HIV, lymphoproliferative malignancies or those undergoing immunosuppressive therapies.^45 46^ Currently, no proven therapeutic strategy for this disease has been established. Based on evidence that T cell exhaustion might affect the course of JCV infections, ICIs are currently tested for treatment of PML.^47^ For example, Cortese *et al* 48 assessed the safety and efficacy of PD-1 inhibition by pembrolizumab with different predisposing conditions (n=8). In this small cohort, five patients achieved a clinical benefit or stabilization of PML and a decrease in JCV viral load. Contrasting results were observed in another study, three kidney transplant recipients suffering from PML were treated with nivolumab and all three patients died within 8 weeks with evidence of disease progression.^49^ As an explanation for this adverse outcome,^48^ the authors speculate that immunosuppressive therapy (ie, calcineurin inhibitors) might have led to persistent T cell dysfunction and lymphopenia. The authors further point out that patients with severe lymphopenia also did not respond favorably to ICI treatment. Hence, they concluded that ICI may be ineffective in such patients.^48 49^ In contrast, a number of case reports describe favorable outcomes for patients with PML receiving ICI. For example, Hoang *et al* 50 described a biopsy-confirmed PML case in which the PD-1 inhibitor nivolumab seems to have stimulated immune activation resulting in effective disease control in the patient with a concomitant hematological malignancy.^50–52^

Together, at present, there are few studies on the safety and/or efficacy of ICI in patients with PML. The available studies do not show consistent results which, might be due to the great heterogeneity of predisposing conditions leading to PML. Despite these difficulties, the present data suggest that the underlying cause of immunosuppression, pretreatment, and laboratory parameters, such as lymphopenia, have relevant effects on the success of ICI therapy of PML. This notion may be extrapolated to ICI treatment of cancer in patients with PML. To the best of our knowledge, however, there exist no definite data on patients with PML receiving ICI treatment because of coexisting cancer. Of course, the use of immunosuppressive comedication for ICI-induced irAEs is challenging in this particular patient population. Thus, prospective studies are necessary to determine whether ICIs are a safe and effective approach for PML and PML-associated cancers.

## Influenza

Influenza, a highly contagious respiratory disease, is responsible for a significant economic burden on the healthcare systems. The co-circulating influenza A (subtype: H1N1, H3N2) and B (lineage: Victoria, Yamagata) viruses cause seasonal epidemics which affect a major part of the global population annually and cause more than 645,000 influenza-associated deaths worldwide.^53 54^ Since patients with cancer are at higher risk of influenza-associated complications, vaccination, the primary preventive tool against influenza, is recommended. Particularly, ICI-treated patients produce robust humoral and cellular immune responses. Still it is important to note that Bersanelli *et al* 53 reported that the post-vaccination occurrence of the influenza syndrome (fever ≥38°C and the presence of at least one respiratory symptom together with generalized symptoms) was significantly increased in patients with cancer receiving ICI. In this study, the lack of efficacy of vaccination was more pronounced among the elderly.^53^ Importantly, influenza vaccination did not negatively impact the efficacy of the anticancer effects of ICI treatment. Moreover, the same authors reported that ICI-treated cancer patients who received influenza vaccination and/or developed influenza syndrome showed longer overall survival. The effect of immunosuppressive therapy for ICI-induced irAE on the influenza syndrome was not addressed in these studies.^54^

In rare cases, influenza infection and vaccination are associated with the occurrence of organ-specific autoimmune conditions, such as Guillain-Barré syndrome, a rapid onset muscle weakness caused by the immune system attacking peripheral nerves. The risk of influenza vaccine-induced Guillain-Barré syndrome is extremely small. Notably, however, Yuen *et al* 55 reported a patient with previous post-vaccination Guillain-Barré syndrome who developed a fatal reactivation following the initiation of ICI. Indeed, the hypothesis that influenza vaccination may induce adverse immune responses in ICI-treated patients is corroborated by results from animal experiments showing increased T cell responses to viral antigens under PD-1 inhibition.^56^ Moreover, it has been suggested that vaccines may stimulate an overwhelming expansion of autoreactive T lymphocytes which cross-recognize vaccines as well as self-antigens.^57 58^

Läubli *et al* 59 reported a small cohort of ICI-treated cancer patients that had received influenza vaccinations and subsequently experienced higher rates of irAEs than expected (n=23). Later studies investigating larger patient cohorts did not confirm such an increase of frequency or severity of irAEs in patients on ICI who previously received influenza vaccination.^57 60–65^ The data of Chong *et al* 57 do not indicate an increase in incidence or severity of irAE in patients with cancer on ICI who received influenza vaccinations. Twenty per cent (75/370) of patients experienced a new irAE (any grade), of those 48% received corticosteroids or other immunosuppressants. In the latter subgroup, no negative influence on vaccination outcome was reported.^57^ In this context, the study of Awadalla *et al* 65 should be pointed out. They actually demonstrated that the administration of a influenza vaccination was not correlated with an increased risk of subsequent myocarditis among patients on ICI. Indeed, rates of vaccination were lower among patients who did develop ICI-induced myocarditis, and the vaccination was associated with a lower risk of other irAEs, in particular ICI-induced pneumonitis.^65^ Unfortunately, there is little data available whether ICI would be beneficial in the management of severe influenza cases. In this context, Yu *et al* 66 reported that influenza infection substantially increases the number of highly PD-1 positive innate lymphoid cells in the lungs of mice. Anti-PD-1 treatment resulted in reduction of total lung innate lymphoid cells with almost complete loss of PD-1 highly positive cells. Hence, they suggested that anti-PD-1 treatment may provide an effective approach for both disease prevention and treatment.^66^

## COVID-19

In March 2020, the COVID-19 outbreak, which is caused by the severe acute respiratory syndrome coronavirus 2 (SARS-COV-2), was officially proclaimed a pandemic by the WHO. Up through end of April, 2020, almost 3 million cases of COVID-19 were confirmed with more than 200,000 deaths reported worldwide.^67^ COVID-19 is predominantly characterized by high fever, dry cough, fatigue, and eventually pneumonia, and can cause death in severe cases. To date, most published data on COVID-19 have been generated in China. In 14% of confirmed cases, the course of disease was severe and in 5% critical. The so-called case fatality rate (CFR) was as high as 1%, thereby being much greater than the CFR usually observed in seasonal influenza (approximately 0.1%). However, current infection rates as well as CFR still have to be considered with caution.^68^ Risk for severe disease and death is strongly associated with older age (in particular >70 years), cardiovascular disease, diabetes, obesity, chronic respiratory disease, hypertension, and cancer.^68–70^ Laboratory predictors for severe and fatal disease predominantly include elevated lactate dehydrogenase, pro-calcitonin, and D-dimers, increased serum levels of cytokines IL-6, IL-10 and tumor necrosis factor-α (TNF-α), as well as decreased lymphocyte counts, particularly CD8+ T and NK cells.^69–72^

Indeed, Biao *et al* 71 recently demonstrated that the number of total T lymphocytes in the peripheral blood was significantly decreased in patients with COVID-19. This was particularly pronounced in older patients and in patients who needed intensive care unit (ICU) treatment. Importantly, lymphopenia was negatively associated with patient survival. Biao *et al* 69–71 also showed that T cell counts of patients who recovered increased, while IL-6, IL-10 and TNF-α levels decreased.^71^ Since the cytokine increases were paralleled by a decrease in lymphocytes, Diao *et al* 71 speculated that elevated proinflammatory cytokines may promote the reduction of T lymphocytes in patients with COVID-19. However, this observation has to be substantiated in future studies.^71^ Additionally, as assessed by flow cytometry of peripheral blood, T cells of severely affected patients are characterized by a much higher PD-1 expression than healthy controls.^71^ Specifically, enhanced PD-1 and Tim-3 expression on T cells was observed when patients progressed from prodromal to symptomatic stages, indicating that T lymphocyte exhaustion—similar to other viral infections—is a hallmark of COVID-19 as well. The expression of critical inhibitory checkpoint proteins of T cell exhaustion, including PD-1 and Tim-3, and the increase of TNF-α, IL-10 and other cytokines (which actually all may modulate the expression of PD-1 and Tim-3) are likely to mediate T cell lymphopenia in patients with COVID-19 through programmed cell death.^73^

To date, very limited data are available addressing the clinical outcome of ICI-treated cancer patients also suffering from COVID-19 infection.^74–77^ The blockade of PD-1+ on CD8+ lymphocytes by ICI treatment might be reasonable in patients with COVID-19 in order to abrogate functional T cell exhaustion and restore vigorous T lymphocytic cytotoxicity against both the tumor and the viral antigens. However, as discussed by Chiappelli *et al*,^75^ this may work only at the initial and intermediate stage when PD-1 expression on cytotoxic T cells ranges between low and medium levels. At the more advanced stage, when PD-1 expression is high on CD8+ T lymphocytes, T lymphocyte exhaustion is likely irreversible, and thus, ICI will no longer have an effect.^75^ The excess of cytokines is also of great significance with respect to the cytokine release syndrome (CRS, ‘cytokine storm’), a phenomenon of massive inflammatory reaction, where cytokines (eg, IL-6, IL-10, TNF-α) are rapidly produced in large amounts in response to infectious agents.^71^ Similar to SARS-COV and the Middle East respiratory syndrome (MERS-COV), SARS-COV-2 is associated with increased amounts of proinflammatory cytokines in the serum, which are suspected to cause pulmonary inflammation and extensive lung damage.^72^ However, unlike SARS-COV and MERS-COV, SARS-COV-2 infection appears to be associated with the activation of both T helper 1 (Th-1) and Th-2 lymphocytes. SARS-COV-2 predominantly targets epithelial cells of the respiratory tract, leading to severe alveolar damage. However, COVID-19 also shows evidence for changes in the lung stroma, suggesting that also pulmonary fibrosis is induced at some time point.^58^ Moreover, similar to SARS-COV and MERS-COV, SARS-COV-2 may also pose the risk of autoimmunity due to cross-reactivity of the induced immune reaction to both viral (eg, spike surface proteins) and host protein epitopes. Lyons-Weiler^58^ recently hypothesized that based on homology with human proteins, such pathogenic priming involving autoimmunity might also occur with SARS-COV-2. Similar to the results of previous SARS-COV animal experiments, Agrawall *et al* 78 reported that mice vaccinated against MERS-COV developed severe Th-2-driven immunopathologies in the lung following post-vaccination MERS-COV challenge.^58 78^ Given the data existing so far on possible autoimmunity particular in the advanced stage of COVID-19, the administration of ICI could pose the risk of immune overactivation and aggravation of autoimmune processes.

Similar to the ‘cytokine storm’ observed in patients with advanced COVID-19, CRS has been observed in rare cases as irAEs in patients receiving ICI.^79 80^ Furthermore, one of the observed irAE of anti-PD-1-based ICI is autoimmune pneumonitis, which may occur in up to 5% of all treated patients.^79–81^ Patients with non-small cell lung cancer treated with ICI may even experience autoimmune pneumonitis in up to 20% of cases.^81^ Since the clinical symptoms and radiographic findings in irAE-induced pneumonitis are similar to those of COVID-19 pneumonitis, it may be in some cases difficult to arrive at proper diagnostic conclusions as the basis for appropriate management. The occurrence of irAEs frequently necessitates the use of systemic corticosteroids or other immunosuppressive/immunomodulating agents such as mycophenolate acid or TNF-α blockers. Whether the use of systemic corticosteroids is harmful in the setting of COVID-19 is unclear. The use of glucocorticoids in patients with SARS-COV-associated and MERS-COV-associated pneumonitis is still controversial because of divergent clinical outcomes reported in the existing literature.^82^ Still, high-dose glucocorticoids are one of the most frequently used adjuncts in acute respiratory distress syndrome, even though the effectiveness of steroids in the management of acute lung injury is ambiguous.^82^ This is also true for COVID-19. Nevertheless, it seems that early and short-time use of low-dose methylprednisolone may be one feasible approach in SARS-COV-2-related pneumonitis and respiratory distress syndrome.^83^ Hence, possible ICI-induced irAEs could be safely managed with methylprednisolone in the setting of COVID-19. Based on the observation of cytokine excess (eg, TNF-α), during advanced stages of COVID-19, one may speculate that TNF-α blockers such as infliximab may not only be beneficial for the management of ICI-induced irAEs, but also for COVID-19.^84^ Notably, cytokine IL-6 antibodies (eg, tocilizumab) are currently under intense investigation in patients with COVID-19. Tocilizumab is already approved in the USA to manage CRS occurring after chimeric antigen receptor T cell treatment.^82^ Notably, it has been demonstrated that tocilizumab is effective in the management of irAEs occurring in ICI-treated patients who are refractory to corticosteroids.^82^ Furthermore, there is currently an ongoing phase II trial investigating a combination of tocilizumab with anti-PD-1/CTLA-4 therapy in order to diminish irAEs in unresectable stage III or stage IV melanoma patients (NCT03999749).^84^

A novel approach to ICI in cancer and viral infections, particularly COVID-19, represents CD200 checkpoint reversal.^85^ The CD200 immune checkpoint causes suppression of the secretion of proinflammatory cytokines, that is, IL-2 and IFN-ү, and enhances the production of myeloid-derived suppressor cells and Tregs that are implicated in impaired antitumor and antiviral immune responses.^85^ Anti-CD200 targeted treatments have shown promising effects in animal models accompanied by downregulation of inhibitory PD-1 receptors.^85^ In a murine coronavirus model, the checkpoint inhibitory CD200-CD200R1 system has been demonstrated to downregulate the single strand RNA virus sensor toll-like receptor seven in myeloid-derived cells and respective interventions restored IFN-ү levels resulting in enhanced virus elimination.^86 87^

Together, experience and evidence are growing with regard to the use of ICI in patients with the novel infectious disease COVID-19. In comparison to chemotherapeutic regimens ICI cannot be considered immunosuppressive. Hematological irAEs caused by ICI are very infrequent, with only few cases reported. In a meta-analysis of 9324 patients, the frequency of neutropenia was smaller than 1%.^88^ Previously reported reactivation of viral infections (ie, cytomegalovirus, hepatitis B) under ICI therapy were mostly observed following immunosuppressive treatment of irAEs. Hence, it does not appear reasonable to assume that patients undergoing ICI are at higher risk of becoming infected by SARS-COV-2 or other infectious agents compared with patients without ICI treatment.^76^

On the basis of preliminary basic research data and clinical observations in patients with COVID-19, one may assume that ICI could safely be employed in cancer patients with a SARS-CoV-2 infection and even COVID-19. As long as there is no clear evidence, however, it remains a case-by-case scenario depending on many factors discussed above, particularly with respect to how advanced the cancer and/or COVID-19 are.^76 79 89–91^ Quaglino *et al* 91 recently reported their experience in 80 melanoma patients who were under ICI at the beginning of March 2020. Quaglino *et al* 91 concluded that their observations give support to the possibility of continuing ICI in melanoma patients (n=80). Accordingly, Luo *et al* 92 observed that PD-1 blockade exposure was not associated with increased risk of severity of COVID-19 in patients with lung cancer.^89 92^ Notably, ICI may even represent an effective approach in the management of COVID-19 patients without cancer.^71 73 76 79^ Additionally, a combination of ICI with an anti-IL-6 antibody is an attractive approach to reduce the risks of both irAEs and possible cytokine excess frequently observed in severe COVID-19 cases.^93^ A study following this strategy is currently recruiting patients. The objective of this prospective, controlled, randomized, multicenter study is to compare the efficacy of the combined administration of a chloroquine analog, nivolumab, and tocilizumab versus standard of care in patients with advanced or metastatic cancer who have COVID-19 and are not eligible to a resuscitation unit (ClinicalTrials.gov: NCT04333914). Patients will be randomized into two different cohorts: (1) asymptomatic or mild symptoms: chloroquine analog versus nivolumab versus standard of care (1:1:1); (2) moderate/severe symptoms: chloroquine analog versus tocilizumab versus standard of care (1:1:1). It has to be stressed, however, that hydroxychloroquine use has been reported to be ineffective or even associated with higher mortality and therefore should only be considered in the context of well-designed controlled and regulatory approved clinical trials. Furthermore, two protocols have been registered at ClinicalTrials.gov investigating ICI in COVID-19 patients without cancer: In a phase II randomized trial, the protocol CORIMUNO19-NIVO will evaluate the efficacy and safety of nivolumab alone versus standard of care in COVID-19 patients hospitalized in an ICU (NCT04343144), and in an open-label, controlled, single-center pilot study, nivolumab will be employed in adult patients with COVID-19 aiming to investigate the efficacy and safety of nivolumab in relation to viral clearance (NCT04356508).

## Conclusions

Based on the data presented in this review and in line with the recommendations of the Study Group for Infections in Compromised Hosts, we assume that PD-1/PD-L1- and/or CTLA-4-based ICI treatment does not seem to independently enhance the risk of infection or cause more virulent course of disease.^27^ Hence, the above discussed viral infections should not be considered as contraindications per se for patients who are scheduled for or are on ICI. Over the course of ICI treatment, however, supportive immunosuppressive therapies may be required to treat ICI-associated irAEs, which in turn may increase the risk of new or reactivation of persisting viral infections. Hence, physicians caring for patients receiving immunosuppressants for treatment of ICI-induced irAEs should maintain close surveillance for the occurrence of symptoms or signs suggestive of new infection or worsening of preexisting viral infections.^27^ For almost all aforementioned viral infections, there are convincing data that disease-associated T cell exhaustion is a fundamental immune escape mechanism. Accordingly, increasing lines of evidence suggest that ICIs represent not only an effective antitumor treatment regimen in such patients but also a potential approach in the management of the viral infection per se.

## References

[1] Darvin P , Toor SM , Sasidharan Nair V , et al . Immune checkpoint inhibitors: recent progress and potential biomarkers. Exp Mol Med 2018;50:1–11. 10.1038/s12276-018-0191-1 PMC6292890 30546008

[2] Zhang N , Tu J , Wang X , et al . Programmed cell death-1/programmed cell death ligand-1 checkpoint inhibitors: differences in mechanism of action. Immunotherapy 2019;11:429–41. 10.2217/imt-2018-0110 30698054

[3] Granier C , De Guillebon E , Blanc C , et al . Mechanisms of action and rationale for the use of checkpoint inhibitors in cancer. ESMO Open 2017;2:e000213. 10.1136/esmoopen-2017-000213 28761757 PMC5518304

[4] Vaddepally RK , Kharel P , Pandey R , et al . Review of indications of FDA-approved immune checkpoint inhibitors per NCCN guidelines with the level of evidence. Cancers 2020;12:738. 10.3390/cancers12030738 PMC7140028 32245016

[5] Qin S , Xu L , Yi M , et al . Novel immune checkpoint targets: moving beyond PD-1 and CTLA-4. Mol Cancer 2019;18:155. 10.1186/s12943-019-1091-2 31690319 PMC6833286

[6] Leung CS , Yang KY , Li X , et al . Single-Cell transcriptomics reveal that PD-1 mediates immune tolerance by regulating proliferation of regulatory T cells. Genome Med 2018;10:71. 10.1186/s13073-018-0581-y 30236153 PMC6148788

[7] Dyck L , Mills KHG . Immune checkpoints and their inhibition in cancer and infectious diseases. Eur J Immunol 2017;47:765–79. 10.1002/eji.201646875 28393361

[8] Wykes MN , Lewin SR . Immune checkpoint blockade in infectious diseases. Nat Rev Immunol 2018;18:91–104. 10.1038/nri.2017.112 28990586 PMC5991909

[9] Hashimoto M , Kamphorst AO , Im SJ , et al . Cd8 T cell exhaustion in chronic infection and cancer: opportunities for interventions. Annu Rev Med 2018;69:301–18. 10.1146/annurev-med-012017-043208 29414259

[10] Johnson DB , Sullivan RJ , Menzies AM . Immune checkpoint inhibitors in challenging populations. Cancer 2017;123:1904–11. 10.1002/cncr.30642 28241095 PMC5445005

[11] Khoja L , Day D , Wei-Wu Chen T , et al . Tumour- and class-specific patterns of immune-related adverse events of immune checkpoint inhibitors: a systematic review. Ann Oncol 2017;28:2377–85. 10.1093/annonc/mdx286 28945858

[12] Wang A , Xu Y , Fei Y , et al . The role of immunosuppressive agents in the management of severe and refractory immune-related adverse events. Asia Pac J Clin Oncol 2020. 10.1111/ajco.13332. [Epub ahead of print: 24 Mar 2020]. 32212243

[13] Chomont N , El-Far M , Ancuta P , et al . Hiv reservoir size and persistence are driven by T cell survival and homeostatic proliferation. Nat Med 2009;15:893–900. 10.1038/nm.1972 19543283 PMC2859814

[14] Sauce D , Almeida JR , Larsen M , et al . Pd-1 expression on human CD8 T cells depends on both state of differentiation and activation status. AIDS 2007;21:2005–13. 10.1097/QAD.0b013e3282eee548 17885290

[15] Hoffmann M , Pantazis N , Martin GE , et al . Exhaustion of activated CD8 T cells predicts disease progression in primary HIV-1 infection. PLoS Pathog 2016;12:e1005661. 10.1371/journal.ppat.1005661 27415828 PMC4945085

[16] Kaufmann DE , Kavanagh DG , Pereyra F , et al . Upregulation of CTLA-4 by HIV-specific CD4+ T cells correlates with disease progression and defines a reversible immune dysfunction. Nat Immunol 2007;8:1246–54. 10.1038/ni1515 17906628

[17] Evans VA , van der Sluis RM , Solomon A , et al . Programmed cell death-1 contributes to the establishment and maintenance of HIV-1 latency. AIDS 2018;32:1491–7. 10.1097/QAD.0000000000001849 29746296 PMC6026054

[18] Velu V , Shetty RD , Larsson M , et al . Role of PD-1 co-inhibitory pathway in HIV infection and potential therapeutic options. Retrovirology 2015;12:14. 10.1186/s12977-015-0144-x 25756928 PMC4340294

[19] Cook MR , Kim C . Safety and efficacy of immune checkpoint inhibitor therapy in patients with HIV infection and advanced-stage cancer: a systematic review. JAMA Oncol 2019;5:1049–54. 10.1001/jamaoncol.2018.6737 30730549

[20] Sorotsky H , Hogg D , Amir E , et al . Characteristics of immune checkpoint inhibitors trials associated with inclusion of patients with HIV: a systematic review and meta-analysis. JAMA Netw Open 2019;2:e1914816. 10.1001/jamanetworkopen.2019.14816 31702796 PMC6902798

[21] Gonzalez-Cao M , Martinez-Picado J , Provencio Pulla M , et al . A phase II exploratory study of durvalumab (MEDI4736) in HIV-1 patients with advanced solid tumors. Ann Oncol 2017;28.

[22] Rajdev L , Chiao EY , Lensing S , et al . Amc 095 (AIDS malignancy Consortium): a phase I study of ipilimumab (IPI) and nivolumab (NIVO) in advanced HIV associated solid tumors (ST) with expansion cohorts in HIV associated solid tumors and classical Hodgkin lymphoma (cHL). JCO 2018;36:TPS2597-TPS:TPS2597. 10.1200/JCO.2018.36.15_suppl.TPS2597

[23] Uldrick TS , Ison G , Rudek MA , et al . Modernizing clinical trial eligibility criteria: recommendations of the American Society of clinical Oncology-Friends of cancer research HIV Working group. J Clin Oncol 2017;35:3774–80. 10.1200/JCO.2017.73.7338 28968173 PMC5793223

[24] Colston E , Grasela D , Gardiner D , et al . An open-label, multiple ascending dose study of the anti-CTLA-4 antibody ipilimumab in viremic HIV patients. PLoS One 2018;13:e0198158. 10.1371/journal.pone.0198158 29879143 PMC5991705

[25] Gay CL , Bosch RJ , Ritz J , et al . Clinical trial of the anti-PD-L1 antibody BMS-936559 in HIV-1 infected participants on suppressive antiretroviral therapy. J Infect Dis 2017;215:1725–33. 10.1093/infdis/jix191 28431010 PMC5790148

[26] Liu J , Pan W , Yang D . The era of immune checkpoint therapy: from cancer to viral Infection-A mini Comment on the 2018 medicine Nobel Prize. Virol Sin 2018;33:467–71. 10.1007/s12250-018-0077-3 30570713 PMC6335215

[27] Redelman-Sidi G , Michielin O , Cervera C , et al . Escmid Study Group for infections in compromised hosts (ESGICH) consensus document on the safety of targeted and biological therapies: an infectious diseases perspective (immune checkpoint inhibitors, cell adhesion inhibitors, sphingosine-1-phosphate receptor modulators and proteasome inhibitors). Clin Microbiol Infect 2018;24 Suppl 2:S95–107. 10.1016/j.cmi.2018.01.030 29427804 PMC5971148

[28] Hoang TN , Paiardini M . Role of cytokine agonists and immune checkpoint inhibitors toward HIV remission. Curr Opin HIV AIDS 2019;14:121–8. 10.1097/COH.0000000000000528 30585798 PMC6469389

[29] Hong CY , Sinn DH , Kang D , et al . Incidence of extrahepatic cancers among individuals with chronic hepatitis B or C virus infection: a nationwide cohort study. J Viral Hepat 2020. 10.1111/jvh.13304. [Epub ahead of print: 27 Apr 2020]. 32340080

[30] Hsu C , Marshall JL , He AR . Workup and management of immune-mediated hepatobiliary pancreatic toxicities that develop during immune checkpoint inhibitor treatment. Oncologist 2020;25:105–11. 10.1634/theoncologist.2018-0162 32043797 PMC7011649

[31] Shah NJ , Al-Shbool G , Blackburn M , et al . Safety and efficacy of immune checkpoint inhibitors (ICIs) in cancer patients with HIV, hepatitis B, or hepatitis C viral infection. J Immunother Cancer 2019;7:353. 10.1186/s40425-019-0771-1 31847881 PMC6918622

[32] Rao M , Valentini D , Dodoo E , et al . Anti-Pd-1/Pd-L1 therapy for infectious diseases: learning from the cancer paradigm. Int J Infect Dis 2017;56:221–8. ‐. 10.1016/j.ijid.2017.01.028 28163164

[33] Pu D , Yin L , Zhou Y , et al . Safety and efficacy of immune checkpoint inhibitors in patients with HBV/HCV infection and advanced-stage cancer: a systematic review. Medicine 2020;99:e19013. 10.1097/MD.0000000000019013 32000444 PMC7004734

[34] Tapia Rico G , Chan MM , Loo KF . The safety and efficacy of immune checkpoint inhibitors in patients with advanced cancers and pre-existing chronic viral infections (hepatitis B/C, HIV): a review of the available evidence. Cancer Treat Rev 2020;86:102011. 10.1016/j.ctrv.2020.102011 32213376

[35] Pertejo-Fernandez A , Ricciuti B , Hammond SP , et al . Safety and efficacy of immune checkpoint inhibitors in patients with non-small cell lung cancer and hepatitis B or hepatitis C infection. Lung Cancer 2020;145:S0169-5002(20)30310-X:181–5. 10.1016/j.lungcan.2020.02.013 32423643

[36] Wieland D , Hofmann M , Thimme R . Overcoming CD8+ T-cell exhaustion in viral hepatitis: lessons from the mouse model and clinical perspectives. Dig Dis 2017;35:334–8. 10.1159/000456584 28468011

[37] Pauken KE , Sammons MA , Odorizzi PM , et al . Epigenetic stability of exhausted T cells limits durability of reinvigoration by PD-1 blockade. Science 2016;354:1160–5. 10.1126/science.aaf2807 27789795 PMC5484795

[38] Hoogeveen RC , Boonstra A . Checkpoint inhibitors and therapeutic vaccines for the treatment of chronic HBV infection. Front Immunol 2020;11:401. 10.3389/fimmu.2020.00401 32194573 PMC7064714

[39] Bengsch B , Seigel B , Ruhl M , et al . Coexpression of PD-1, 2B4, CD160 and KLRG1 on exhausted HCV-specific CD8+ T cells is linked to antigen recognition and T cell differentiation. PLoS Pathog 2010;6:e1000947. 10.1371/journal.ppat.1000947 20548953 PMC2883597

[40] Gardiner D , Lalezari J , Lawitz E , et al . A randomized, double-blind, placebo-controlled assessment of BMS-936558, a fully human monoclonal antibody to programmed death-1 (PD-1), in patients with chronic hepatitis C virus infection. PLoS One 2013;8:e63818. 10.1371/journal.pone.0063818 23717490 PMC3661719

[41] El-Khoueiry AB , Sangro B , Yau T , et al . Nivolumab in patients with advanced hepatocellular carcinoma (CheckMate 040): an open-label, non-comparative, phase 1/2 dose escalation and expansion trial. Lancet 2017;389:2492–502. 10.1016/S0140-6736(17)31046-2 28434648 PMC7539326

[42] Gane E , Verdon DJ , Brooks AE , et al . Anti-Pd-1 blockade with nivolumab with and without therapeutic vaccination for virally suppressed chronic hepatitis B: a pilot study. J Hepatol 2019;71:900–7. 10.1016/j.jhep.2019.06.028 31306680

[43] Liu J , Zhang E , Ma Z , et al . Enhancing virus-specific immunity in vivo by combining therapeutic vaccination and PD-L1 blockade in chronic hepadnaviral infection. PLoS Pathog 2014;10:e1003856. 10.1371/journal.ppat.1003856 24391505 PMC3879364

[44] Tan CS , Koralnik IJ . Progressive multifocal leukoencephalopathy and other disorders caused by JC virus: clinical features and pathogenesis. Lancet Neurol 2010;9:425–37. ‐. 10.1016/S1474-4422(10)70040-5 20298966 PMC2880524

[45] Weber T , Trebst C , Frye S , et al . Analysis of the systemic and intrathecal humoral immune response in progressive multifocal leukoencephalopathy. J Infect Dis 1997;176:250–95. 10.1086/514032 9207375

[46] Kartau M , Sipilä JO , Auvinen E , et al . Progressive multifocal leukoencephalopathy: current insights. Degener Neurol Neuromuscul Dis 2019;9:109–21. 10.2147/DNND.S203405 31819703 PMC6896915

[47] Koralnik IJ . Can immune checkpoint inhibitors keep JC virus in check? N Engl J Med 2019;380:1667–8. 10.1056/NEJMe1904140 30969502 PMC8656172

[48] Cortese I , Muranski P , Enose-Akahata Y , et al . Pembrolizumab treatment for progressive multifocal leukoencephalopathy. N Engl J Med 2019;380:1597–605. 10.1056/NEJMoa1815039 30969503

[49] Medrano C , Vergez F , Mengelle C , et al . Effectiveness of immune checkpoint inhibitors in transplant recipients with progressive multifocal leukoencephalopathy. Emerg Infect Dis 2019;25:2145–7. 10.3201/eid2511.190705 31625858 PMC6810212

[50] Hoang E , Bartlett NL , Goyal MS , et al . Progressive multifocal leukoencephalopathy treated with nivolumab. J Neurovirol 2019;25:284–7. 10.1007/s13365-019-00738-x 30864100 PMC6506384

[51] Audemard-Verger A , Gasnault J , Faisant M , et al . Sustained response and rationale of programmed cell Death-1-Targeting for progressive multifocal leukoencephalopathy. Open Forum Infect Dis 2019;6:ofz374. 10.1093/ofid/ofz374 31660340 PMC6767972

[52] Tan CS , Bord E , Broge TA , et al . Increased program cell death-1 expression on T lymphocytes of patients with progressive multifocal leukoencephalopathy. J Acquir Immune Defic Syndr 2012;60:244–8. 10.1097/QAI.0b013e31825a313c 22549384 PMC3400136

[53] Bersanelli M , Giannarelli D , Castrignanò P , et al . Influenza vaccine indication during therapy with immune checkpoint inhibitors: a transversal challenge. The INVIDIa study. Immunotherapy 2018;10:1229–39. 10.2217/imt-2018-0080 30326787

[54] Bersanelli M , Buti S , De Giorgi U , et al . State of the art about influenza vaccination for advanced cancer patients receiving immune checkpoint inhibitors: when common sense is not enough. Crit Rev Oncol Hematol 2019;139:87–90. 10.1016/j.critrevonc.2019.05.003 31112887

[55] Yuen C , Kamson D , Soliven B , et al . Severe relapse of vaccine-induced Guillain-Barré syndrome after treatment with nivolumab. J Clin Neuromuscul Dis 2019;20:194–9. 10.1097/CND.0000000000000230 31135622

[56] Finnefrock AC , Tang A , Li F , et al . Pd-1 blockade in rhesus macaques: impact on chronic infection and prophylactic vaccination. J Immunol 2009;182:980–7. 10.4049/jimmunol.182.2.980 19124741

[57] Chong CR , Park VJ , Cohen B , et al . Safety of inactivated influenza vaccine in cancer patients receiving immune checkpoint inhibitors. Clin Infect Dis 2020;70:193–9. 10.1093/cid/ciz202 30874791 PMC6938975

[58] Lyons-Weiler J . Pathogenic priming likely contributes to serious and critical illness and mortality in COVID-19 via autoimmunity. J Transl Autoimmun 2020;3:100051. 10.1016/j.jtauto.2020.100051 32292901 PMC7142689

[59] Läubli H , Balmelli C , Kaufmann L , et al . Influenza vaccination of cancer patients during PD-1 blockade induces serological protection but may raise the risk for immune-related adverse events. J Immunother Cancer 2018;6:40. 10.1186/s40425-018-0353-7 29789020 PMC5964701

[60] Gwynn ME , DeRemer DL , Saunders KM , et al . Immune-Mediated adverse events following influenza vaccine in cancer patients receiving immune checkpoint inhibitors. J Oncol Pharm Pract 2020;26:647–54. 10.1177/1078155219868758 31474214

[61] Wijn DH , Groeneveld GH , Vollaard AM , et al . Influenza vaccination in patients with lung cancer receiving anti-programmed death receptor 1 immunotherapy does not induce immune-related adverse events. Eur J Cancer 2018;104:182–7. 10.1016/j.ejca.2018.09.012 30368069

[62] Failing JJ , Ho TP , Yadav S , et al . Safety of influenza vaccine in patients with cancer receiving pembrolizumab. JCO Oncol Pract 2020;6:JOP1900495:JOP.19.00495. 10.1200/JOP.19.00495 PMC8462591 32048920

[63] Groeneveld GH , Wijn DH , Vollaard AM . Immune-Related adverse events in patients with cancer receiving influenza vaccination and immune checkpoint inhibitors. Clin Infect Dis 2020;70:1519. 10.1093/cid/ciz512 31210266 PMC7076744

[64] Keam B , Kang CK , Jun KI , et al . Immunogenicity of influenza vaccination in patients with cancer receiving immune checkpoint inhibitor. Clin Infect Dis 2019:pii: ciz1092. 10.1093/cid/ciz1092 31680143

[65] Awadalla M , Golden DLA , Mahmood SS , et al . Influenza vaccination and myocarditis among patients receiving immune checkpoint inhibitors. J Immunother Cancer 2019;7:53. 10.1186/s40425-019-0535-y 30795818 PMC6387531

[66] Yu Y , Tsang JCH , Wang C , et al . Single-cell RNA-seq identifies a PD-1^hi^ ILC progenitor and defines its development pathway. Nature 2016;539:102–6. 10.1038/nature20105 27749818

[67] COVID-19 Dashboard by the center for systems science and engineering (CSSE) at Johns Hopkins University (JHU). Available: https://coronavirus.jhu.edu/map.html [Accessed 26 Apr 2020].

[68] Jordan RE , Adab P , Cheng KK . Covid-19: risk factors for severe disease and death. BMJ 2020;368:m1198. 10.1136/bmj.m1198 32217618

[69] Zhou F , Yu T , Du R , et al . Clinical course and risk factors for mortality of adult inpatients with COVID-19 in Wuhan, China: a retrospective cohort study. Lancet 2020;395:1054–62. 10.1016/S0140-6736(20)30566-3 32171076 PMC7270627

[70] Li X , Xu S , Yu M , et al . Risk factors for severity and mortality in adult COVID-19 inpatients in Wuhan. J Allergy Clin Immunol 2020;pii: S0091-6749:30495–4. 10.1016/j.jaci.2020.04.006 PMC7152876 32294485

[71] Diao B , Wang C , Tan Y , et al . Reduction and functional exhaustion of T cells in patients with coronavirus disease 2019 (COVID-19). Front Immunol 2020;11:827. 10.3389/fimmu.2020.00827 32425950 PMC7205903

[72] Huang C , Wang Y , Li X , et al . Clinical features of patients infected with 2019 novel coronavirus in Wuhan, China. Lancet 2020;395:497–506. 10.1016/S0140-6736(20)30183-5 31986264 PMC7159299

[73] Chiappelli F , Khakshooy A , Greenberg G . CoViD-19 immunopathology and immunotherapy. Bioinformation 2020;16:219–22. 10.6026/97320630016219 32308263 PMC7147500

[74] Bonomi L , Ghilardi L , Arnoldi E , et al . A rapid fatal evolution of coronavirus Disease-19 in a patient with advanced lung cancer with a long-time response to nivolumab. J Thorac Oncol 2020;15:e83–5. 10.1016/j.jtho.2020.03.021 32243919 PMC7270552

[75] Bersanelli M , Buti S , De Giorgi U , et al . State of the art about influenza vaccination for advanced cancer patients receiving immune checkpoint inhibitors: when common sense is not enough. Crit Rev Oncol Hematol 2019;139:87–90. 10.1016/j.critrevonc.2019.05.003 31112887

[76] Kattan J , Kattan C , Assi T . Do checkpoint inhibitors compromise the cancer patients' immunity and increase the vulnerability to COVID-19 infection? Immunotherapy 2020;12:351–4. 10.2217/imt-2020-0077 32290754 PMC7161588

[77] Rassy E , Khoury-Abboud R-M , Ibrahim N , et al . What the oncologist needs to know about COVID-19 infection in cancer patients. Future Oncol 2020;16:1153–6. 10.2217/fon-2020-0312 32323577 PMC7192200

[78] Agrawal AS , Tao X , Algaissi A , et al . Immunization with inactivated middle East respiratory syndrome coronavirus vaccine leads to lung immunopathology on challenge with live virus. Hum Vaccin Immunother 2016;12:2351–6. 10.1080/21645515.2016.1177688 27269431 PMC5027702

[79] Bersanelli M . Controversies about COVID-19 and anticancer treatment with immune checkpoint inhibitors. Immunotherapy 2020;12:imt-2020-0067 10.2217/imt-2020-0067 32212881 PMC7117596

[80] Rotz SJ , Leino D , Szabo S , et al . Severe cytokine release syndrome in a patient receiving PD-1-directed therapy. Pediatr Blood Cancer 2017;64:e26642. 10.1002/pbc.26642 28544595

[81] Suresh K , Voong KR , Shankar B , et al . Pneumonitis in non-small cell lung cancer patients receiving immune checkpoint immunotherapy: incidence and risk factors. J Thorac Oncol 2018;13:1930–9. 10.1016/j.jtho.2018.08.2035 30267842

[82] Qin Y-Y , Zhou Y-H , Lu Y-Q , et al . Effectiveness of glucocorticoid therapy in patients with severe coronavirus disease 2019: protocol of a randomized controlled trial. Chin Med J 2020;133:1080-1086. 10.1097/CM9.0000000000000791 32149773 PMC7147272

[83] Zheng C , Wang J , Guo H , et al . Risk-adapted treatment strategy for COVID-19 patients. Int J Infect Dis 2020;94:74–7. 10.1016/j.ijid.2020.03.047 32229257 PMC7270846

[84] Ascierto PA , Fox BA , Urba WJ , et al . Insights from immuno-oncology: the Society for immunotherapy of cancer statement on access to IL-6-targeting therapies for COVID-19. J Immunother Cancer 2020;8:e000878. 10.1136/jitc-2020-000878 32300051 PMC7204613

[85] Xiong Z , Ampudia Mesias E , Pluhar GE , et al . Cd200 checkpoint reversal: a novel approach to immunotherapy. Clin Cancer Res 2020;26:232–41. 10.1158/1078-0432.CCR-19-2234 31624103

[86] Ceribelli A , Motta F , De Santis M , et al . Recommendations for coronavirus infection in rheumatic diseases treated with biologic therapy. J Autoimmun 2020;109:102442. 10.1016/j.jaut.2020.102442 32253068 PMC7127009

[87] Karnam G , Rygiel TP , Raaben M , et al . Cd200 receptor controls sex-specific TLR7 responses to viral infection. PLoS Pathog 2012;8:e1002710. 10.1371/journal.ppat.1002710 22615569 PMC3355091

[88] Zhuang J , Du J , Guo X , et al . Clinical diagnosis and treatment recommendations for immune checkpoint inhibitor-related hematological adverse events. Thorac Cancer 2020;11:799–804. 10.1111/1759-7714.13281 32017466 PMC7049514

[89] Davis AP , Boyer M , Lee JH , et al . COVID-19: the use of immunotherapy in metastatic lung cancer. Immunotherapy 2020;12:545–8. 10.2217/imt-2020-0096 32349579 PMC7202359

[90] Vardhana SA , Wolchok JD . The many faces of the anti-COVID immune response. J Exp Med 2020;217:pii: e20200678. 10.1084/jem.20200678 PMC7191310 32353870

[91] Quaglino P , Fava P , Brizio M , et al . Metastatic melanoma treatment with checkpoint inhibitors in the COVID‐19 era: experience from an Italian skin cancer unit. J Eur Acad Dermatol Venereol 2020;38. 10.1111/jdv.16586 PMC7276756 32426877

[92] Luo J , Rizvi H , Egger JV , et al . Impact of PD-1 blockade on severity of COVID-19 in patients with lung cancers. Cancer Discov 2020:CD-20-0596. 10.1158/2159-8290.CD-20-0596 PMC7416461 32398243

[93] Bonam SR , Kaveri SV , Sakuntabhai A , et al . Adjunct immunotherapies for the management of severely ill COVID-19 patients. Cell Rep Med 2020;1:100016. 10.1016/j.xcrm.2020.100016 32562483 PMC7190525

